# Adherence to antiretroviral therapy for HIV/AIDS in Latin America and the Caribbean: Systematic review and meta‐analysis

**DOI:** 10.1002/jia2.25066

**Published:** 2018-01-22

**Authors:** Jessica de Mattos Costa, Thiago Silva Torres, Lara Esteves Coelho, Paula Mendes Luz

**Affiliations:** ^1^ Instituto Nacional de Infectologia Evandro Chagas Fundação Oswaldo Cruz Rio de Janeiro Brazil

**Keywords:** anti‐HIV agents, antiretroviral therapy, Caribbean region, developing countries, highly active, Latin America, medication adherence

## Abstract

**Introduction:**

Optimal adherence to antiretroviral therapy is closely related with suppression of the HIV viral load in plasma, slowing disease progression and decreasing HIV transmission rates. Despite its importance, the estimated proportion of people living with HIV in Latin America and the Caribbean with optimal adherence has not yet been reported in a meta‐analysis. Moreover, little is known of the factors leading to poor adherence which may be setting‐specific. We present a pooled estimate of adherence to antiretroviral therapy (ART) of people living with HIV in Latin America and Caribbean, report the methods used to measure adherence and describe the factors associated with poor adherence among the selected studies.

**Methods:**

We electronically searched published studies up to July 2016 on the PubMed, Web of Science and Virtual Health Library (Latin America and the Caribbean Regional Portal); considering the following databases: MEDLINE, LILACS, PAHO and IBECS. Two independent reviewers selected and extracted data on ART adherence and study characteristics. Pooled estimate of adherence was derived using a random‐effects model. Risk of bias in individual studies was assessed independently by two investigators using the Risk of Bias Assessment tool for Non‐randomized Studies (RoBANS).

**Results and discussion:**

The meta‐analysis included 53 studies published between 2005 and 2016, which analysed 22,603 people living with HIV in 25 Latin America and Caribbean countries. Overall adherence in Latin America and Caribbean was 70% (95% CI: 63–76; *I* ^*2*^ = 98%), similar to levels identified by studies conducted in high‐income regions. Self‐report was the most frequently used method to measure adherence. Subgroup analysis showed that adherence was higher for the shortest recall time frame used, as well as in countries with lower income level, Gross National Income (GNI) per capita and Human Development Index (HDI). Studies reported diverse adherence barriers, such as alcohol and substance misuse, depression, unemployment and pill burden.

**Conclusions:**

Our study suggests that adherence to ART in Latin America and Caribbean may be below the sufficient levels required for a successful long‐term viral load suppression.

**Abbreviations**
ACTG: AIDS Clinical Trials Group
ART: antiretroviral therapy
CAT‐VIH: *Cuestionario de Adherencia al Tratamiento para el VIH/SIDA*
CEAT‐VIH: *Cuestionario para la Evaluación de la Adhesión al Tratamiento antirretroviral*
GNI: gross national income
HDI: human development index
LAC: Latin America and the Caribbean
PLHIV: people living with HIV
PMAQ: Patient Medication Adherence Questionnaire
PRISMA: preferred reporting items for systematic reviews and meta‐Analyses
RCT: randomized controlled trial
RoBANS: risk of bias assessment tool for non‐randomized studies
SMAQ: Simplified Medication Adherence Questionnaire
VAS: visual analogue scale
VPAD‐24: *Variables psicológicas y comportamientos de adhesión*

## 1 Introduction

Latin America and the Caribbean (LAC) consists of 33 sovereign countries which cover an area that stretches from the northern border of Mexico to the southern tip of Chile, including the Caribbean. It has an area of over 20 million km^2^, as of 2017, its population was estimated at approximately 650 million (~9% of the world population), being predominantly urban (80%) 1. LAC is mostly a developing region which had a combined nominal gross domestic product (GDP) of 5,5 trillion USD and a GDP purchasing power parity (PPP) of 9.7 trillion USD in 2017 2. By 2015, the region's HDI was 0.731 (high), varying from 0.493 (Haiti, low) to 0.847 (Chile, very high) 3.

Antiretroviral therapy (ART) revolutionized the treatment of people living with HIV (PLHIV) by dramatically decreasing their morbidity and mortality 4. In LAC region there were 2.1 million PLHIV and more than 1.1 million PLHIV on ART by the end of 2016 5. Indeed, studies have indicated promising results in the region with regards to the HIV Care Continuum: clinical retention, ART use and viral suppression significantly improved from 2003 to 2012 (63 to 77%, 74 to 91% and 53 to 82% respectively; *p* < 0.05, each), though disparities for vulnerable groups, such as female sex workers, people who inject drugs, gay men and other men who have sex with men, remain 6.

Argentina, Bolivia, Brazil, Costa Rica, Mexico, Paraguay, Uruguay and Venezuela, in Latin America; and Antigua and Barbuda, Bahamas, Barbados, Saint Vincent and the Grenadines, and Trinidad and Tobago, in the Caribbean, have now adopted the World Health Organization recommendation of initiation of ART for all PLHIV irrespective of CD4 cell count 7. ART coverage reaches 58% (95% confidence interval [95%CI] 42–72%) and 52% (95%CI 41–60%) of all PLHIV in LAC, respectively 7. At the country level, treatment coverage was 70% in Cuba, followed by 64% in Argentina, 62% in Trinidad and Tobago and 60% in Brazil and Mexico. On the other hand, Bolivia had only 25%, Paraguay had 35% and Guatemala had 36% of PLHIV accessing treatment in 2016 5. From 2010 to 2016, the number of deaths has not dramatically decreased (12% of decrease in Latina America and 28% in the Caribbean) and, most significantly, the number of new infections remained relatively stable (0% of decrease in Latin America and 5% decrease in the Caribbean) 5. This stability hides big differences between countries. In Latin America, although new infections have decreased by more than 20% in Colombia, El Salvador, Nicaragua and Uruguay, they increased significantly in Chile (34%), followed by Guatemala (23%), Costa Rica (16%), Honduras (11%) and Panama (9%); and slightly in Argentina and Brazil (3%). In the Caribbean, the majority of new infections occurred in Cuba, where estimated numbers of new HIV infections more than doubled between 2010 and 2016 7, despite of the dramatic increase in treatment coverage 5.

The Political Declaration on Ending AIDS by 2030 8 established specific goals for LAC region, which included reducing the number of new infections in LAC from 100,000 to 40,000 and increasing the number of PLHIV on ART from 1.1 to 1.6 million by 2020. Achievement of these goals will be challenging, requiring continued efforts from governments and international agencies.

Specifically, the HIV epidemic cannot be ended without containing the new infections, and adherence to ART plays an important role in this process. ART adherence is closely linked to suppression of the HIV viral load in plasma 9, 10 which leads to immune reconstitution and also decreases onward HIV transmission 11. The optimal adherence level to achieve viral suppression is unclear, though the 95% threshold established by Paterson et al. has largely been used as a goal 9. More recently, other authors demonstrated that high levels of viral suppression could be obtained with adherence levels below 95% when in use of newer ART regimens 12, 13, 14, 15, 16. The monitoring of ART adherence is highly recommended by health organizations 17 and the main methods are: self‐report by interview, pill counts, pharmacy refill and medication event monitoring system (MEMS). Monitoring patient's ART adherence is a challenging but critical way to identify those with poor adherence.

Even though there is a huge difference in socio‐cultural characteristics across countries in LAC, there are still similar inequalities and traditional values that may act as barriers for HIV treatment, which may impact adherence to ART. This is more pronounced among high‐risk populations, who are more vulnerable to social inequalities, discrimination and violence. However, little is known of the factors leading to poor adherence which may be setting‐specific. Although ART coverage has increased in the region, the fact that the number of new infections remained relatively stable may be related to the lack of adherence to ART.

In this meta‐analysis, we synthesize the published peer reviewed literature, generating a pooled estimate of adherence to ART of PLHIV in LAC. In addition, we present the adherence proportion according to the country's income level, Human Development Index (HDI) rank, and Gross National Income (GNI) per capita, as well as other factors as detailed in the Methods section. We qualitatively synthetize the methods used to monitor adherence and describe the factors associated with poor adherence among the selected studies. Greater knowledge of ART adherence levels of PLHIV in LAC may provide means to improve patient care and could help Governments and regional institutions to accomplish the goal of ending AIDS by 2030.

## 2 Methods

This systematic review and meta‐analysis has been reported according to the *Preferred reporting items for systematic reviews and meta‐Analyses* (PRISMA) *Statement* 18.

### 2.1 Protocol and registration

Key information about the design and conduct of this systematic review and meta‐analysis are recorded at the international database of prospectively registered systematic reviews in health and social care (PROSPERO 2017:CRD42017055963) 19.

### 2.2 Eligibility criteria

Studies of any design were included if they met all the following criteria: (i) the study involved people living with HIV/AIDS in Latin America and the Caribbean (studies involving participants from other regions were included if we could clearly identify data from LAC participants); (ii) participants were receiving antiretroviral therapy; (iii) and treatment adherence was quantified. Studies were excluded if they included pregnant women and did not stratified adherence for the non‐pregnant participants, because of specific features of their ART (treatment aimed at preventing vertical transmission of HIV and not as treatment of infection). Also, studies were excluded if they assessed alternative forms of treatment (for example, due to some specific co‐infection), or if antiretrovirals were being used for post or pre‐exposure prophylaxis. Similarly, we excluded studies focusing only on participants less than 18 years of age or specific populations (for example, only individuals previously found to have low adherence, or homeless populations). Studies including both adults and participants less than 18 years of age were not excluded if adherence data was stratified by age (and in this case, data from the age categories <18 years were not considered in the present analysis). Articles published before 2005 were excluded to avoid studies in the pre‐HAART era. Also, grey literature was not considered in this study (such as thesis, dissertations, monographies, conference papers and reports).

### 2.3 Information sources and search strategy

Articles were identified through searches conducted on 14 July 2016 on PubMed, Web of Science and Virtual Health Library (Latin America and the Caribbean Regional Portal) considering the following databases: MEDLINE, LILACS, PAHO and IBECS. The search combined terms derived from four domains: (a) adherence; (b) HIV; (c) antiretroviral (d) countries of Latin America and the Caribbean (see Additional file 1 for the full PubMed search strategy). Citations were inserted into the study database when the four domains were jointly present in the title, abstract, MeSH terms or keywords. No limits were applied for language or publication date in the search. A reference manager (Zotero) was used to collect and organize search results and for duplicate removal.

### 2.4 Study selection

Two investigators (JMC, TST) reviewed all abstracts and full‐text articles independently, according to the eligibility criteria. Discrepancies were adjudicated by an independent third investigator (PML).

### 2.5 Data collection process

Data extraction was performed independently by two investigators (JMC, TST) using a predefined extraction form. Each paper was coded for publication characteristics (authors, publication year, full title, journal and language), study characteristics (years when data was collected, country(ies) where the study was performed, study design, sample size, recruitment setting and number of study centres), participants characteristics (age, sex and race/ethnicity), adherence monitoring characteristics (method for adherence measurement, cutoff of optimal adherence, proportion of adherents, time frame used to measure adherence), and the factors significantly associated with adherence (*p *<* *0.05) on multivariate modelling. Discrepancies in extracted data were adjudicated by an independent third investigator (PML).

### 2.6 Study definitions

Adherence was estimated for each study by dividing the number of individuals with optimal adherence by the number of individuals evaluated. This implies that our overall pooled adherence was based on the adherence threshold adopted in each study. When a study examined the effect of an intervention on ART adherence, only the adherence result at baseline was considered. In case there was no baseline assessment, only the first adherence assessment of the control group was extracted and analysed. As adopted in prior studies 20, 21 when more than one adherence measurement was reported, the most objective method was chosen for the analysis (e.g. medication event monitoring system (MEMS) > pill count > pharmacy refill > self‐reported adherence in the past week > self‐reported adherence in the past month). When an optimal adherence threshold (e.g. ≥80 or ≥95%) was not explicitly defined in the study and adherence was categorized in levels (for example, low, regular and strict; or 65**–**84%, 85**–**94% and 95**–**100%), the highest adherence category was considered.

For subgroup analysis, adherence recall time frame was categorized in four periods: 3–4 days, 7 days, 30 days and 90 days. Location/country was classified by geographical area and categorized as: Brazil, South America (Chile, Colombia, Peru), Central America and Caribbean (Cuba, Dominican Republic, Guatemala, Haiti, Jamaica), North America (Puerto Rico and Mexico) and multi‐region (included countries from more than one region). Time period when study was conducted was categorized as ≤2005, 2006**–**2010 and ≥2011. Study design was categorized as cross‐sectional, longitudinal (non‐RCT) and RCT. Income group was categorized by low/lower middle, upper middle, high and mix, following the World Bank definitions for 2017 22. HDI and GNI per capita data were extracted from the Human Development Reports of the United Nations Development Programme 3. The HDI is a composite index measuring average achievement in three basic dimensions of human development ‐ a long and healthy life, knowledge and a decent standard of living 3; while the GNI per capita reflects the average income of a country's citizens 3. HDI ranking and the GNI per capita, were classified in two categories each (HDI: <0.754 and ≥0.754; GNI per capita in USD: <14,145 and ≥14,145). When a study involved multiple countries, the lower HDI or GNI value was considered, though we repeated the analysis considering the highest HDI and GNI values and the results changed minimally. Number of study sites was categorized as single‐site, multi‐site and online (participants accessed and completed survey via an online portal). Treatment experience was categorized as naïve (included individuals who had just recently started ART, usually within six months), experienced (included individuals who had been using ART for some time) and naïve and experienced. Instrument used to measure adherence were categorized as self‐report, MEMS, self‐report+drug refill and self‐report+pill count. Adherence threshold was categorized as <94% (when threshold was ≥64%, ≥80%, ≥85% or ≥90%), 95% (when threshold was ≥95%), 100% and not reported. Finally, the presence of statistical models evaluating factors associated with adherence (YES/NO) was evaluated in subgroup analysis.

### 2.7 Risk of bias in individual studies

The quality of the included studies was assessed by two investigators (PML, LEC) using the Risk of Bias Assessment tool for Non‐randomized Studies (RoBANS) 23. RoBANS includes criteria for judging the risk of bias for each domain. The risk of bias in a study was graded as low, high or unclear based on the following study features: selection of participants (selection bias), consideration of confounding variables (selection bias), measurement of exposure (detection bias), handling of incomplete outcome data (attrition bias) and selective outcome reporting (reporting bias).

### 2.8 Data synthesis

Logit transformation of the proportions and their standard errors were calculated to achieve a normal distribution which is required for the pooling of data 24. Pooled adherence proportion was calculated using the DerSimonian‐Laird method 25 assuming a random‐effects model. Heterogeneity between studies was initially evaluated by visual inspection of forest‐plots. The proportion of true heterogeneity to total variance was calculated by the Higgins *I* ^2^ statistic 26.

### 2.9 Additional analyses

We conducted subgroup analyses and estimated the pooled adherence proportion according to adherence recall time frame, location/country, time period when study was conducted, study design, country's income level, HDI rank, GNI per capita, sites, treatment experience, instrument to measure adherence, adherence threshold, and presence of statistical models evaluating factors associated with adherence.

## 3 Results

### 3.1 Study Characteristics

**Figure 1:**
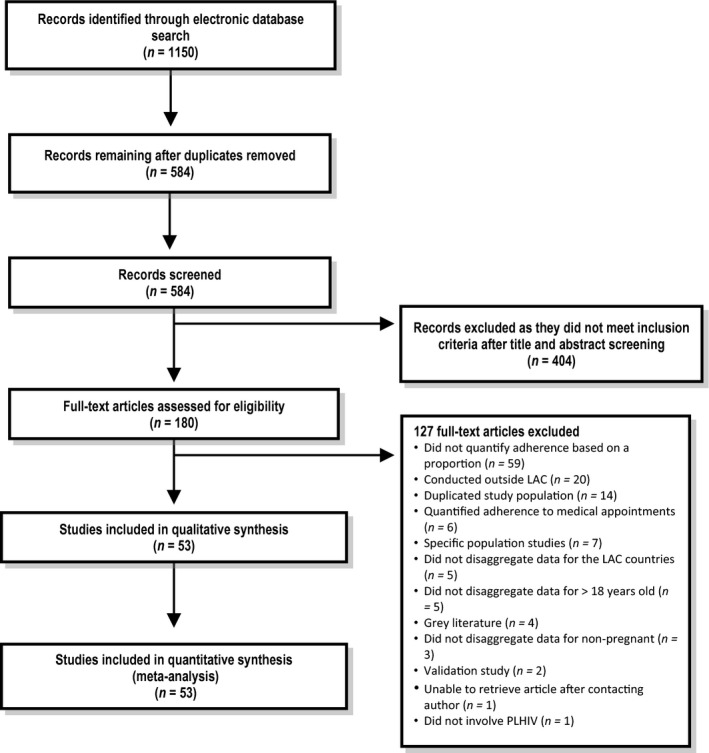
Flow diagram of study selection for the meta‐analysis of adherence to antiretroviral therapy for HIV/AIDS in Latin America and the Caribbean, 2005–2016. LAC, Latin America and the Caribbean; PLHIV = people living with HIV.

The flow diagram of study selection is shown in Figure 1. Fifty‐three studies, composed of 22,603 participants in ART from 25 LAC countries, met eligibility criteria for the systematic review (Table 1) 27, 28, 29, 30, 31, 32, 33, 34, 35, 36, 37, 38, 39, 40, 41, 42, 43, 44, 45, 46, 47, 48, 49, 50, 51, 52, 53, 54, 55, 56, 57, 58, 59, 60, 61, 62, 63, 64, 65, 66, 67, 68, 69, 70, 71, 72, 73, 74, 75, 76, 77, 78, 79. The median number of participants in ART per study was 201 [range: 13**–**3343; interquartile range (IQR): 394**–**125]. The studies were conducted between 2000 and 2013 and published from 2005 to 2016 in three different languages (English: 39; Spanish: 9; Portuguese: 5). Studies were mostly conducted in countries with an upper middle income level (81.1%).

**Table 1:** Characteristics of studies included in the meta‐analysis of adherence to antiretroviral therapy for HIV/AIDS in Latin America and the Caribbean, 2005–2016

Source	LAC country	Study design	N in analysis	Adherence measure (instrument)	Optimal adherence threshold (%)	Recall time frame (days)	Country's income group^a^	HDI^b^	GNI per capita^b^
Alave et al., 2013 48	Peru	Non‐RCT longitudinal	1478	SR	>95	30	Upper middle	0.74	11,295
Allen et al., 2011 38	Antigua and Barbuda, Grenada, Trinidad and Tobago	Cross‐sectional	274	SR	≥95	7	Upper middle and high	0.754–0.786	11,502–28,049
Amico et al., 2005 29	Puerto Rico	Cross‐sectional	196	SR (modified ACTG)	≥95	3	High	Not available	Not available
Aragonés et al., 2011 39	Cuba	Cross‐sectional	781	SR	≥95	7	Upper middle	0.775	7455
Arrivillaga et al., 2009 36	Colombia	Cross‐sectional	269	SR	≥64	Not reported	Upper middle	0.727	12,762
Balandrán et al., 2013 56	Mexico	Cross‐sectional	2054	SR (ACTG)	≥95	4	Upper middle	0.762	16,383
Basso et al., 2013 55	Brazil	RCT	108	MEMS	≥95	30	Upper middle	0.754	14,145
Biello et al., 2016 78	17 countries^c^	Cross‐sectional	1637	SR	100	30	Lower middle, upper middle and high	0.625–0.847	4466 –21,665
Bonolo et al., 2005 27	Brazil	Non‐RCT longitudinal	306	SR	≥95	3	Upper middle	0.754	14,145
Calvetti et al., 2014 57	Brazil	Cross‐sectional	120	SR (CEAT‐VIH)	Not reported	Not reported	Upper middle	0.754	14,145
Campbell et al., 2010 37	Guatemala	Cross‐sectional	122	SR (VAS) > Pill count	≥95	7	Lower middle	0.64	7063
Cardona‐Arias et al., 2011 40	Colombia	Cross‐sectional	146	SR (SMAQ)	Not reported	Not reported	Upper middle	0.727	12,762
Carrillo et al., 2009 35	Colombia	Cross‐sectional	103	SR	Not reported	Not reported	Upper middle	0.727	12,762
Carvalho et al., 2007 32	Brazil	Non‐RCT longitudinal	150	SR	≥95	7	Upper middle	0.754	14,145
Casotti et al., 2011 41	Brazil	Cross‐sectional	81	SR (CEAT‐VIH)	≥85	Not reported	Upper middle	0.754	14,145
Costa et al., 2012 19	Brazil	RCT	13	MEMS > Pill count > SR	>95	30	Upper middle	0.754	14,145
De Boni et al., 2016 77	6 countries^d^	Cross‐sectional	3343	SR	Not reported	7	Lower middle, upper middle and high	0.625–0.847	4466–21,665
De La Hoz et al., 2014 63	Colombia	Cross‐sectional	122	SR	≥80	Not reported	Upper middle	0.727	12,762
Drachler et al., 2016 75	Brazil	Non‐RCT longitudinal	267	SR	≥95	30	Upper middle	0.754	14,145
Ferro et al., 2015 64	Peru	Cross‐sectional	263	SR (VAS)	≥90	30	Upper middle	0.74	11,295
Fleming et al., 2016 79	Dominican Republic	Cross‐sectional	21	SR	100	Not reported	Upper middle	0.722	12,756
Garcia et al., 2006 30	Brazil	Cross‐sectional	182	SR (modified PMAQ)	>95	90	Upper middle	0.754	14,145
Gutierrez et al., 2012 47	Brazil	Cross‐sectional	284	SR	100	3, 7	Upper middle	0.754	14,145
Hanif et al., 2013 49	Brazil	Cross‐sectional	632	SR (modified ACTG)	100	4	Upper middle	0.754	14,145
Harris et al., 2011 42	Dominican Republic	Cross‐sectional	300	SR (VAS)	≥95	30	Upper middle	0.722	12,756
Ilias et al., 2011 43	Brazil	Cross‐sectional	56	SR	≥80	3	Upper middle	0.754	14,145
Ivers et al., 2014 58	Haiti	RCT	488	SR	100	30	Low	0.493	1657
Jacques et al., 2014 59	Brazil	Cross‐sectional	152	SR (CEAT‐VIH)	>85	Not reported	Upper middle	0.754	14,145
Krishnan et al., 2015 65	Peru	Cross‐sectional	313	SR (VAS)	≥90	Not reported	Upper middle	0.74	11,295
Magidson et al., 2015 66	17 countries^e^	Cross‐sectional	2211	SR	100	30	Lower middle, upper middle and high	0.625–0.847	4466–21,665
Magidson et al., 2016 74	Brazil	Cross‐sectional	182	SR	Not reported	90	Upper middle	0.754	14,145
Malbergier et al., 2015 67	Brazil	Cross‐sectional	438	SR (SMAQ)	Not reported	7	Upper middle	0.754	14,145
Malow et al., 2013 50	Haiti	Cross‐sectional	194	SR	Not reported	Not reported	Low	0.493	1657
Mascolini et al., 2008 33	6 countries^f^	Cross‐sectional	592	SR	Not reported	30	Upper middle and high	0.722–0.827	8350**–**20,945
Muñoz et al., 2011 44	Peru	Non‐RCT longitudinal	60	SR (ACTG)	≥95	30	Upper middle	0.74	11,295
Nachega et al., 2012 46	Brazil	Cross‐sectional	201	SR (ACTG)	100	30	Upper middle	0.754	14,145
Pacífico et al., 2015 73	Peru	Cross‐sectional	364	SR (SMAQ)+Withdrawal^g^	Not reported	Not reported	Upper middle	0.74	11,295
Padoin et al., 2013 51	Brazil	Cross‐sectional	125	SR	100	7	Upper middle	0.754	14,145
Pérez‐Salgado et al., 2015 68	Mexico	Cross‐sectional	557	SR	>95	7,30	Upper middle	0.762	16,383
Piña López et al., 2008 34	Mexico	Cross‐sectional	64	SR (VPAD‐24)	100	30	Upper middle	0.762	16,383
Remien et al., 2007 31	Brazil	Cross‐sectional	200	SR (modified ACTG)	≥90	3	Upper middle	0.754	14,145
Santillán Torres Torija et al., 2015 69	Mexico	Cross‐sectional	109	SR (modified ACTG)	100	30	Upper middle	0.762	16,383
Santos et al., 2005 28	Brazil	Cross‐sectional	394	SR	Not reported	Not reported	Upper middle	0.754	14,145
Silva et al., 2014 60	Brazil	Cross‐sectional	314	SR (CEAT‐VIH)	≥85	Not reported	Upper middle	0.754	14,145
Silveira et al., 2014 62	Brazil	RCT	332	SR	≥95	3	Upper middle	0.754	14,145
Souza et al., 2016 76	Brazil	Cross‐sectional	140	SR (CEAT‐VIH) > Withdrawal	Not reported	7	Upper middle	0.754	14,145
Teixeira et al., 2013 52	Brazil	Non‐RCT longitudinal	144	Pill count+SR (ACTG)^h^	≥95	Not reported	Upper middle	0.754	14,145
Tello‐Velásquez et al., 2015 70	Peru	Cross‐sectional	389	SR (CEAT‐VIH)	Not reported	Not reported	Upper middle	0.74	11,295
Tietzmann et al., 2013 53	Brazil	Cross‐sectional	453	SR	≥95	3	Upper middle	0.754	14,145
Tufano et al., 2015 71	Brazil	Cross‐sectional	438	SR (SMAQ)	Not reported	7, 90	Upper middle	0.754	14,145
Varela et al., 2014 61	Chile	Cross‐sectional	120	SR (Morisky‐Green‐Levine)	Not reported	Not reported	High	0.847	21,665
Varela‐Arévalo et al., 2013 54	Colombia	Cross‐sectional	127	SR (CAT‐VIH)	>90	Not reported	Upper middle	0.727	12,762
Zulliger et al., 2015 72	Dominican Republic	Cross‐sectional	194	SR (ACTG)	100	4	Upper middle	0.722	12,756

ACTG, Aids Clinical Trials Group; CAT‐VIH, *Cuestionario de adherencia al tratamiento para el VIH/SIDA*; CEAT‐VIH, *Cuestionario para la Evaluación de la Adhesión al Tratamiento Antirretroviral*; MEMS, medication event monitoring system; PMAQ, Patient Medication Adherence Questionnaire; RCT, randomized clinical trials; SMAQ, Simplified Medication Adherence Questionnaire; SR, self‐report; VAS, visual analogue scale;

a Study countries were categorized according to the income group, as defined by the World Bank for 2017 22.

b Study countries were categorized according to the United Nations Human Development Index (HDI) ranking and the Gross National Income (GNI) per capita (based on purchasing power parity in constant 2011 international dollars), as defined by the United Nations Development Programme 3.

c Argentina, Bolivia, Brazil, Chile, Colombia, Costa Rica, Ecuador, El Salvador, Guatemala, Honduras, Mexico, Nicaragua, Panama, Paraguay, Peru, Uruguay and Venezuela.

d Argentina, Brazil, Chile, Honduras, Mexico and Peru.

e Argentina, Bolivia, Brazil, Chile, Colombia, Costa Rica, Ecuador, El Salvador, Guatemala, Honduras, Mexico, Nicaragua, Panama, Paraguay, Peru, Uruguay and Venezuela.

f Argentina, Brazil, Dominican Republic, Jamaica, Mexico and Puerto Rico.

g Used two methods to measure adherence, self‐report or medication withdrawal, to calculate study proportion of participants in optimal adherence.

h Used two methods to measure adherence, self‐report and pill count, to calculate study proportion of participants in optimal adherence.

Adherence was most commonly self‐reported via structured interviews (96.2%). Forty‐nine studies (92.4%), enrolling 21,974 participants, provided a self‐reported adherence proportion. Forty‐seven studies (88.7%) used self‐report instruments only and one used MEMS only. Five studies (9.4%) used a combination of patient self‐report, MEMS, pill count and drug refill. The following standardized instruments were used to measure self‐reported adherence: the AIDS Clinical Trials Group (ACTG) adherence instrument 80; the CAT‐VIH ‐ *Cuestionario de adherencia al tratamiento para el VIH/SIDA* 81; the CEAT‐VIH ‐ *Cuestionario para la Evaluación de la Adhesión al Tratamiento Antirretroviral* 82; the Morisky, Green & Levine Medication Adherence Scale 83; the PMAQ ‐ Patient Medication Adherence Questionnaire 84; the SMAQ ‐ Simplified Medication Adherence Questionnaire 85 and the VPAD‐24 ‐ *Variables psicológicas y comportamientos de adhesión* 86. Twenty‐five studies (47.2%) did not report the instrument used or the instrument was designed for the study or adapted from other studies.

Two studies combined two different adherence measures reporting the overall optimal adherence proportion: Teixeira et al. (2013)^52^ (ACTG questionnaire and pill count) and Pacífico et al. (2015)^73^ (SMAQ questionnaire or drug refill). Balandrán et al. (2013) 56, assessed adherence using the ACTG questionnaire (5 items) and the adherence index, but only the results for the ACTG questionnaire were considered in this meta‐analysis. Though as a general rule we opted to use reported data from the most objective methods, a few exceptions were made. Campbell et al. (2010) 37 used both self‐report (Visual Analogue Scale [VAS]) and pill count for measuring adherence. Although pill count was the most objective measure, the time frame information for it was not available, thus only data from the seven‐day recall self‐report measure (VAS) was considered in the analysis. In Souza et al. (2016) 76, only the self‐reported adherence measure was considered because the adherence as measured from medication dispensing data addressed a period greater than three months (the whole study period).

Studies used different thresholds to define optimal adherence (range: ≥64% to > 100%). The most common definitions used for optimal adherence were higher than 95% and 100% of prescribed doses (54.7%). Adherence recall time frames varied between the last three days and the last 90 days. Seventeen studies (32.1%) did not clearly report the time frame used.

Twenty‐four out of 53 studies evaluated factors associated with adherence using adjusted statistical models. Statistically significant factors (*p* < 0.05) associated with adherence to ART found by these studies are presented in Table 2. Some factors positively associated with adherence to ART were: high social support 32, 49, good relationship with the physician 39, 68; satisfaction with the healthcare service 32, 72; and use of counselling services 38. Some factors negatively associated with adherence to ART were: alcohol use or alcohol use disorders 27, 38, 52, 64, 77; substance use 52, 72, 77, 78; high pill burden 27, 47, 62; depression symptoms 61, 62, 71; unemployment or irregular employment 27, 62; and high or detectable HIV viral load 62, 71.

**Table 2:** Factors associated with adherence to antiretroviral therapy for HIV/AIDS in Latin America and the Caribbean, for 24 studies with available data, 2005–2016

Source	Factors associated with adherence
Allen et al., 2011 38	Use of a counselling service (AOR** = **3.20; 95% CI: 1.55**–**6.61; *p *=* *0.002) Revelation of HIV status without consent (AOR** = **2.31; 95% CI: 1.13**–**4.74; *p *=* *0.023) Alcohol consumption (AOR** = **0.47; 95% CI: 0.23**–**0.96; *p *=* *0.039) Side effects (AOR** = **0.32; 95% CI: 0.15**–**0.68; *p* ** = **0.003)
Aragonés et al., 2011 39	Communication with the physician (AOR** = **1.457; 95% CI: 1.010**–**2.103; *p* ** = **0.044) Change in treatment (AOR** = **1.597; 95% CI: 1.083**–**2.358; *p* ** = **0.018) Memory (AOR** = **3.175; 95% CI: 2.112**–**4.774; *p *=* *0.000) Self‐efficacy (AOR** = **2.976; 95% CI: 1.999**–**4.433; *p* ** = **0.000) Commitment to treatment (AOR** = **1.597; 95% CI: 1.093**–**2.334; *p* ** = **0.016) Confidence in treatment (AOR** = **1.817; 95% CI: 1.245**–**2.650; *p* ** = **0.002)
Arrivillaga et al., 2009 36	Membership in the subsidized national health care plan^a^ or being uninsured (AOR** = **3.478; 95% CI: 1.957–6.181; *p *<* *0.0001) when compared to the contributive plan.
Biello et al., 2016 78	Age (AOR** = **1.02; 95% CI: 1.00**–**1.03; *p* ** = **0.04) Hard drug use during sex (AOR** = **0.72; 95% CI: 0.53**–**0.96; *p* ** = **0.03)
Bonolo et al., 2005 27	(Nonadherence) Unemployment (ARH** = **2.17; 95% CI: 1.19–3.96; *p* ** = **0.011) Alcohol use (ARH** = **2.27; 95% CI: 1.58–3.25; *p *<* *0.001) Self‐report of three or more adverse reactions (ARH** = **1.64; 95% CI: 1.09–2.48; *p* ** = **0.017) Number of pills per day (ARH** = **2.04; 95% CI: 1.11–3.76; *p* ** = **0.02) Switch in antiretroviral regimen (ARH** = **2.72; 95% CI: 1.84–4.03; *p *<* *0.001) Use of more than one health service (RH** = **0.54; 95% CI: 0.36–0.80; *p *=* *0.002) Longer time between HIV test and 1st prescription (ARH** = **2.27; 95% CI: 1.52–3.40; *p *<* *0.001)
Calvetti et al., 2014 57	Social class (middle) (AOR** = **3.5250; 95% CI: 1.229**–**10.080; *p* ** = **0.019) Perceived HIV stage (symptomatic) (AOR** = **0.346; 95% CI: 0.138**–**0.871; *p* ** = **0.024) WHOQOL‐HIV bref^b^ domain I/physical^c^ (AOR** = **1.276; 95% CI: 1.010**–**1.613; *p* ** = **0.041) WHOQOL‐HIV bref^b^ domain V/environment^c^ (AOR** = **1.415; 95% CI: 1.158**–**1.728; *p* ** = **0.001)
Carvalho et al., 2007 32	(Nonadherence) Lower educational level (AOR** = **18.4; 95% CI: 2.9**–**118.8; *p* ** = **0.002) Profession (AOR** = **0.2; 95% CI: 0.0**–**0.9; *p* ** = **0.047) Income (AOR** = **1.0; 95% CI: 1.0**–**1.0; *p* ** = **0.007) High social support (AOR** = **10.6; 95% CI: 1.4**–**79.1; *p* ** = **0.022) Satisfaction with the service at the pharmacy (AOR** = **32.5; 95% CI: 4.6**–**227.9; *p *=* *0.000) Healthcare reference centre in *Plano Piloto* (an urban planned location vs. unplanned) (AOR** = **0.2; 95% CI: 0.1**–**0.7; *p* ** = **0.014)
Casotti et al., 2011 41	Higher educational level (AOR** = **1.40; 95% CI: 1.10**–**1.78; *p* ** = **0.006) longer duration of undetectable viral load (AOR** = **1.03; 95% CI: 1.00**–**1.06; *p* ** = **0.02)
De Boni et al., 2016 77	(Nonadherence ‐ missed doses) Substance use (*p *<* *0.001): alcohol use compared to no substance use (AOR** = **2.46; 95% CI: 1.99–3.05) illicit drug use compared to no substance use (AOR** = **3.57; 95% CI: 2.02–6.30) using both alcohol and illicit drugs compared to no substance use (AOR** = **4.98; 95% CI: 3.19–7.79) HIV transmission mode (*p *<* *0.001): homosexual vs. heterosexual (AOR** = **0.88; 95% CI: 0.67**–**1.16) IDU vs. heterosexual (AOR** = **2.46; 95% CI: 1.04**–**5.83) others vs. heterosexual (AOR** = **1.44; 95% CI: 1.05**–**1.98) Age (per ten years increase) (AOR** = **0.88; 95% CI: 0.80**–**0.98; *p *=* *0.02) Study site (AOR** = **1.87; 95% CI: 1.17–3.01 for IHSS/HE‐Honduras vs. FH‐Argentina AOR** = **0.08; 95% CI: 0.04–0.16 for INCMNSZ‐Mexico vs. FH‐Argentina; *p *<* *0.001)
Drachler et al., 2016 75	(Nonadherence) SEA‐ART^d^ score (per each unit increase) (AOR** = **0.92; 95% CI: 0.90**–**0.95; *p *=* *0.002)
Ferro et al., 2015 64	Having an alcohol use disorder with optimal adherence (AOR** = **0.427; 95% CI: 0.187–0.976; *p *=* *0.044) Having an alcohol use disorder with perfect adherence (AOR** = **0.552; 95% CI: 0.327–0.930; *p *=* *0.026)
Gutierrez et al., 2012 47	Having symptoms prior to ART (*p *=* *0.039) Taking fewer ART pills (*p *=* *0.003) Not missing medical appointments (*p *<* *0.0001)
Hanif et al., 2013 49	Having one child (compared to 0 or ≥2) (AOR** = **2.29; 95% CI: 1.33**–**3.94; *p *=* *0.003) High social support (AOR** = **2.85; 95% CI: 1.50**–**5.41; *p* ** = **0.001) High asset index (AOR** = **2.47; 95% CI: 1.79**–**3.40; *p* ** = **0.000) Gender female (AOR** = **0.58; 95% CI: 0.38**–**0.88; *p* ** = **0.011)
Pérez‐Salgado et al., 2015 68	(Low adherence) Patient dissatisfaction about relationship with the physician (AOR** = **1.90; 95% CI: 1.01**–**3.57; *p* ** = **0.046)
Piña López et al., 2008 34	The combination of intermediate levels of stress associated with tolerance to ambiguity and low levels of depression (*p* ** = **0.027)
Remien et al., 2007 31	(Nonadherence) Sexual orientation (heterosexual vs. homosexual/bisexual) (AOR** = **2.69; 95% CI: 1.08–6.66; *p *<* *0.05) Difficulty to tailoring therapeutic regimen to daily routine (AOR** = **2.56; 95% CI: 1.07–6.14; *p *<* *0.05) Loss of appetite in the last month (AOR** = **3.56; 95% CI: 1.31–9.62; *p *<* *0.05)
Silveira et al., 2014 62	No regular employment (ARR** = **0.91; 95% CI: 0.82–1.00; *p* ** = **0.05) Detectable plasma viral load (ARR** = **0.83; 95% CI: 0.73–0.95; *p* ** = **0.01) Depressive symptoms (ARR** = **0.99; 95% CI: 0.99–1.00; *p* ** = **0.04) Number of tablets daily (ARR** = **0.96; 95% CI: 0.93–0.98; *p *<* *0.01)
Teixeira et al., 2013 52	Intensity of alcohol use (AOR** = **3.29; 95% CI: 1.83–5.92; *p *<* *0.001) Use of alcohol and multiple substances (AOR** = **5.99; 95% CI: 1.78–20.28; *p* ** = **0.004)
Tello‐Velásquez et al., 2015 70	(Nonadherence) Moderate/severe poor quality of sleep (APR** = **1.34; 95% CI: 1.17**–**1.54; *p *=* *0.001)
Tietzmann et al., 2013 53	Gender male (APR** = **1.37; 1.24**–**1.52; *p* ** = **0.000) Low and moderate clinical status (compared to severe) (APR** = **1.18; 95% CI: 1.04**–**1.35; *p* ** = **0.009)
Tufano et al., 2015 71	Nonadherence in last seven days: Age in years (AOR** = **0.96; 95% CI: 0.93**–**0.98; *p *<* *0.01) Hamilton Depression Rating Scale (AOR** = **1.04; 95% CI: 1.01**–**1.07; *p *<* *0.01) Viral load (AOR** = **1.21; 95% CI: 1.03**–**1.42; *p *<* *0.05) Nonadherence in last 90 days: Age in years (AOR** = **1.02; 95% CI: 1.00**–**1.05; *p *<* *0.05) Viral load (AOR** = **1.21; 95% CI: 1.03**–**1.42; *p *<* *0.05) Heterosexual HIV transmission mode (compared to homo/bisexual) (AOR** = **0.52; 95% CI: 0.28**–**0.96; *p *<* *0.05) Unknown HIV transmission mode (compared to homo/bisexual) (AOR** = **0.10; 95% CI: 0.01**–**0.88; *p *<* *0.05) CD4 + cell count (AOR** = **0.99; 95% CI: 0.99**–**1.00; *p *<* *0.05)
Varela et al., 2014 61	Nonadherence: Moderate‐severe depressive symptoms [Exp(B) ** = **3.08; 95% CI: 1.08**–**8.80; *p* ** = **0.023]
Varela‐Arévalo et al., 2013 54	Barriers to treatment (AOR** = **7.9; 95% CI: 2.04**–**30.59; *p* ** = **0.003) Men with no family member with HIV (AOR** = **0.10; 95% CI: 0.01**–**0.73; *p* ** = **0.023) Women with no family member with HIV (AOR** = **0.05; 95% CI: 0.00**–**0.73; *p* ** = **0.028)
Zulliger et al., 2015 72	Nonadherence: ‘Female sex worker’‐related discrimination (AOR** = **3.24; 95% CI: 1.28**–**8.20; *p *≤* *0.05) Use of any drug (AOR** = **2.41; 95% CI: 1.09**–**5.34; *p *≤* *0.01) Worked in a ‘Female sex worker’ establishment (AOR** = **2.35; 95% CI: 1.20**–**4.60; *p *≤* *0.05) Internalized stigma related to female sex work (AOR** = **1.09; 95% CI: 1.02**–**1.16; *p *≤* *0.05) Positive perceptions of HIV providers (AOR** = **0.91; 95% CI: 0.85**–**0.98; *p *≤* *0.05)

AOR, adjusted odds ratio; APR, adjusted prevalence ratio; ARR, adjusted relative risk; ARH, adjusted relative hazard; IDU, injection drug use.

a General System of Social Security in Health (*Sistema General de Seguridad Social en Salud*, SGSSS ‐ Colombia).

b WHOQOL‐HIV bref is a shorter version of the original instrument WHOQOL‐HIV, a multi‐dimensional instrument designed to assess the quality of life of people infected with human immunodeficiency virus (HIV).

c Domain I of WHOQOL‐HIV bref includes physical pain, physical problem, energy and sleep quality; and domain V includes physical safety, housing, finance, care (access to quality health care and social services), information, leisure time, physical environment (pollution/noise/transit/climate) and transport.

d The scale of Self‐efficacy Expectations of Adherence to Antiretroviral Treatment (SEA‐ART) assesses patients’ expectations of their own ability to follow the antiretroviral prescription in 21 high‐risk situations for non‐adherence to ART.

### 3.2 Risk of Bias

**Figure 2:**
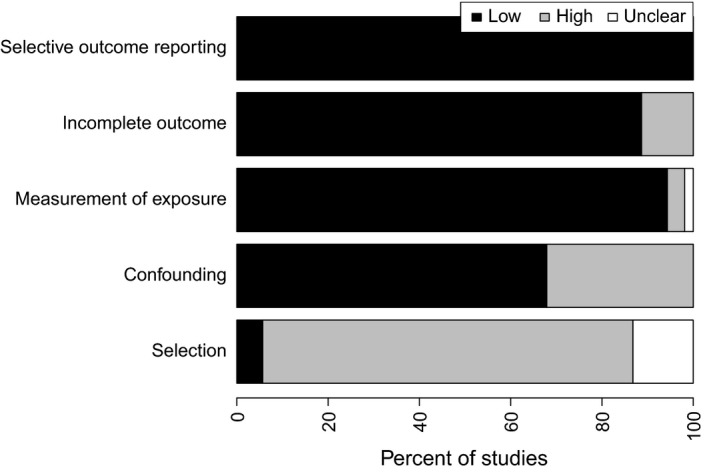
Risk of bias of studies included in the meta‐analysis of adherence to antiretroviral therapy for HIV/AIDS in Latin America and the Caribbean, 2005–2016, presented as percentages across all included studies.

The results of the risk of bias assessment for each study included in the meta‐analysis are shown in Figure 2. The risk of selection biases due to the inadequate selection of participants was high in 43 studies, low in three and unclear in seven. The risk of selection biases due to the inadequate confirmation and consideration of confounding variables during the design and analysis phases was low in 36 studies, but high in the remaining 17. The risk of performance biases caused by inadequate measurements of exposure was low in 50 studies, high in two and unclear in one. The risk of attrition biases caused by the inadequate handling of incomplete outcome data was low in 47 studies and high in the remaining six. The risk of selective reporting bias was low in all studies.

### 3.3 Meta‐analysis

**Figure 3:**
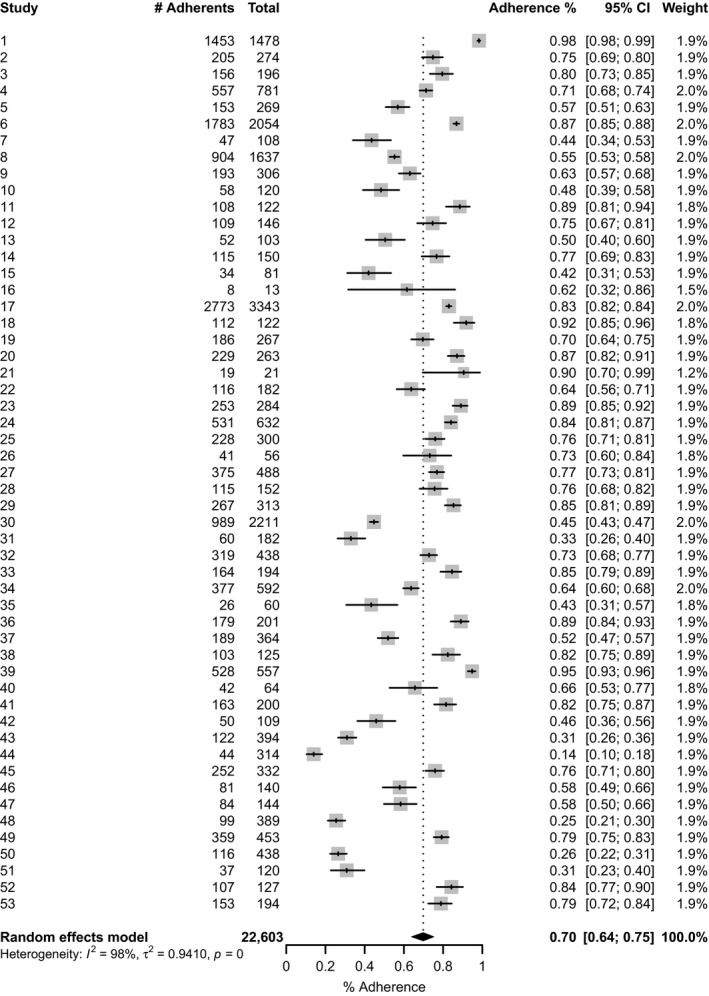
Pooled proportion of PLHIV adhering to antiretroviral therapy in Latin America and Caribbean, 2005–2016. CI, confidence interval; *I* ^*2*^, the percentage of total variation across studies that is due to heterogeneity rather than chance; τ^2^, tau‐squared is an estimate of the between‐study variance; *p*,* p*‐value.

**Figure 4:**
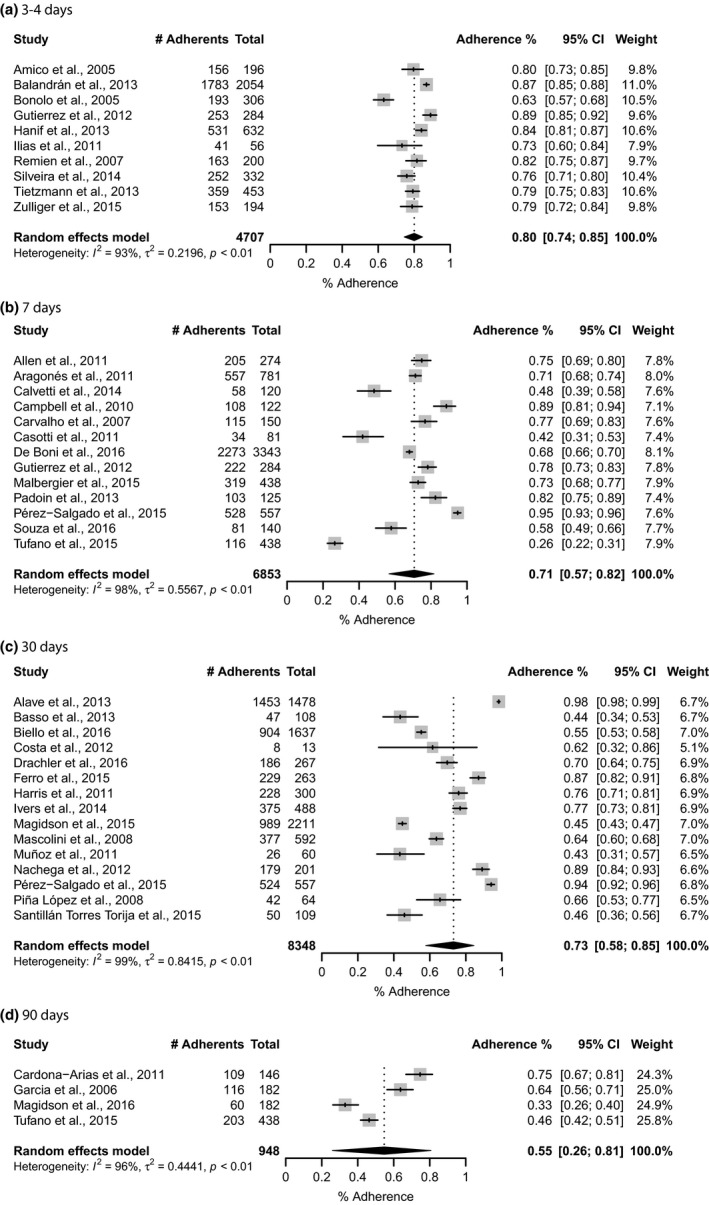
Pooled proportion of PLHIV adhering to ART in LAC by adherence recall time frame, 2005–2016. (a) 3–4 days; (b) seven days; (c) 30 days, (d) 90 days. CI, confidence interval; *I* ^*2*^, the percentage of total variation across studies that is due to heterogeneity rather than chance; τ^2^, tau‐squared is an estimate of the between‐study variance; *p*,* p*‐value.

**Figure 5:**
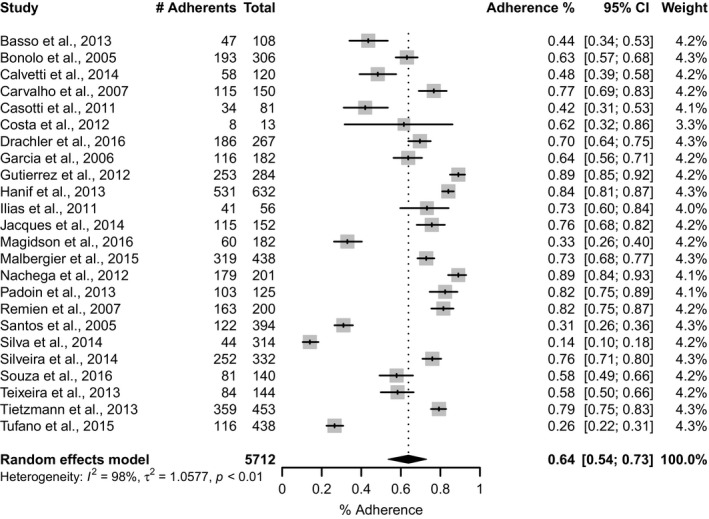
Pooled proportion of PLHIV adhering to ART in Brazil, 2005–2016. CI, confidence interval; *I* ^*2*^, the percentage of total variation across studies that is due to heterogeneity rather than chance; τ^2^, tau‐squared is an estimate of the between‐study variance; *p*,* p*‐value.

The overall pooled adherence was estimated in 70% (95% CI: 63**–**76; *I* ^*2*^ **= **98%) (Figure 3). Results differed when we stratified studies by the four pre‐defined time frames: last 3**–**4 days, last 7 days, last 30 days and last 90 days. The pooled estimate for the shortest period was significantly 7 higher and somewhat less heterogeneous (80%; 95% CI: 74**–**85; *I* ^*2*^ **=** 93%) than for the longest period (55%; 95% CI: 26**–**81; *I* ^*2*^ **= **96%) (Figure 4). We also recalculated the pooled proportion according to the location/country, and in Brazil, where most of studies were conducted (45.3%), the adherence estimate was 64% (95% CI: 54**–**73; *I* ^2^ **= **98%) (Figure 5).

Results of the subgroup analysis are shown in Table 3. Studies conducted in low or lower middle income countries showed a higher pooled adherence (83%; 95% CI: 63**–**93; *I* ^*2*^ **= **81%) than in middle income countries (70%; 95% CI: 62**–**77; *I* ^*2*^ **= **98%). In countries with a lower HDI (<0.754), pooled adherence was higher (75%; 95% CI:64**–**84; *I* ^*2*^ **= **99%) than in countries with a higher HDI (66%; 95% CI: 57**–**74; *I* ^*2*^ **= **98%). Similarly, in countries with a lower GNI per capita (<$ 14,145) the pooled adherence was higher (75%; 95% CI: 65**–**83; *I* ^*2*^ **= **99%) than in countries with a higher GNI per capita (65%; 95% CI: 55**–**74; *I* ^*2*^ **= **98%). Studies addressing only ART naïve participants had lower pooled adherence (56%; 95% CI: 33**–**78; *I* ^*2*^ **= **75%) than those including treatment experienced participants (69%; 95% CI: 62**–**75; *I* ^*2*^ **= **98%). The pooled proportion of adherence for studies using patient's self‐report was 71% (95% CI: 64**–**77; *I* ^*2*^ **= **99%), quite similar to the overall results as expected given that self‐report was the most frequent tool used to measure adherence (96.2%).

**Table 3:** Subgroup analysis of studies included in the meta‐analysis of adherence to antiretroviral therapy for HIV/AIDS in Latin America and the Caribbean, 2005–2016

Analysis group	No of Studies	Sample size	Pooled Adherence (95% CI)	Tests for Heterogeneity	
				*p‐*value (Q Statistic)^f^	*I* ^*2*^ (%)
Overall	53	22603	0.70 (0.64, 0.75)	<0.01	98
Time frame					
3**–**4 days	10	4707	0.80 (0.74, 0.85)	<0.01	93
7 days	13	6853	0.71 (0.57, 0.82)	<0.01	98
30 days	15	8348	0.73 (0.58, 0.85)	<0.01	99
90 days	4	948	0.55 (0.26, 0.81)	<0.01	96
Location/country					
Brazil	24	5712	0.64 (0.54, 0.73)	<0.01	98
SA (Chile, Colombia, Peru)	12	3754	0.71 (0.49, 0.87)	<0.01	99
CA/Caribbean (Cuba, DR, Guatemala, Haiti, Jamaica)	7	2100	0.79 (0.73, 0.85)	<0.01	80
NA (Mexico, Puerto Rico)	5	2980	0.79 (0.47, 0.94)	<0.01	98
Multi‐site	5	8057	0.66 (0.44, 0.82)	<0.01	100
Study period					
≤2005	5	1396	0.68 (0.39, 0.87)	<0.01	98
2006**–**2010	24	7328	0.71 (0.60, 0.79)	<0.01	97
≥2011	12	10025	0.66 (0.45, 0.82)	<0.01	99
Study design					
Cross‐sectional	43	19257	0.69 (0.62, 0.76)	<0.01	99
Longitudinal	6	2405	0.75 (0.38, 0.94)	<0.01	98
RCT	4	941	0.66 (0.39, 0.86)	<0.01	94
Country's income level^a^					
Low/Lower middle	3	804	0.83 (0.63, 0.93)	<0.01	81
Upper middle	43	13426	0.70 (0.62, 0.77)	<0.01	98
High	2^c^				
Mix	5	8057	0.66 (0.44, 0.82)	<0.01	100
HDI^b^					
<0.754	21	12736	0.75 (0.64, 0.84)	<0.01	99
≥0.754	31	9671	0.66 (0.57, 0.74)	<0.01	98
GNI per capita^b^					
<14145	23	13791	0.75 (0.65, 0.83)	<0.01	99
≥14145	29	8616	0.65 (0.55, 0.74)	<0.01	98
Sites					
Single	27	6579	0.65 (0.52, 0.76)	<0.01	98
Multi	23	11585	0.77 (0.70, 0.82)	<0.01	96
Online	3	4440	0.55 (0.31, 0.76)	<0.01	98
Treatment experience					
Naïve	3	510	0.56 (0.33, 0.78)	<0.01	75
Experienced	48	20594	0.69 (0.62, 0.75)	<0.01	98
Naïve and experienced	2^c^				
Instrument to measure adherence					
Self‐report	49	21974	0.71 (0.64, 0.77)	0.02	99
MEMS	2^c^				
Self‐report+Withdrawal^d^	1^c^				
Self‐report+Pill Count^e^	1^c^				
Adherence threshold					
<94%	10	1897	0.72 (0.51, 0.86)	<0.01	98
95%	18	7777	0.77 (0.66, 0.85)	<0.01	97
100%	11	5966	0.75 (0.62, 0.84)	<0.01	98
Not reported	14	6963	0.53 (0.40, 0.66)	<0.01	99
Statistical models evaluating factors associated with adherence					
Yes	24	11425	0.70 (0.60, 0.78)	<0.01	98
No	29	11178	0.70 (0.60, 0.79)	<0.01	99

CA, Central America countries; CI, confidence interval; DR, Dominican Republic; GNI, Gross National Income; HDI, United Nations human development index; MEMS, medication event monitoring system; NA, not applicable to SA or CA; RCT, randomized clinical trials; SA, South American countries.

a Study countries were categorized according to the income group, as defined by the World Bank for 2017 22.

b Study countries were categorized according to the United Nations Human Development Index (HDI) ranking and the Gross National Income (GNI) per capita (based on purchasing power parity in constant 2011 international dollars), as defined by the United Nations Development Programme 3. When a study involved multiple countries, the lower HDI or GNI value was considered.

c When the number of studies in each group was ≤2, meta‐analysis was not performed.

d Used two methods to measure adherence, self‐report or medication withdrawal, to calculate study proportion of participants in optimal adherence.

e Used two methods to measure adherence, self‐report and pill count, to calculate study proportion of participants in optimal adherence.

f *p*‐value for the Q statistic hypothesis test of whether there is heterogeneity, a *p*‐value <0.05 implies a rejection of the null hypothesis that the studies are homogeneous.

## 4 Discussion

This is the first systematic review and meta‐analysis that estimates a pooled proportion of adherence to ART in LAC, uniting evidence from 53 studies, 22,603 participants, in 25 countries. Results suggest that overall, 70% (95% CI: 63**–**76) of PLHIV in LAC were adherent to ART and, consequently, that 30% of PLHIV in LAC may be at risk of developing AIDS‐related illnesses and transmitting the virus to others because they cannot achieve sufficient adherence to ART as required for successful viral load suppression. Mills et al. (2006) 87, in a meta‐analysis of adherence to ART in sub‐Saharan Africa (27 studies; 12,116 participants) and Mhaskar et al. (2013) in a meta‐analysis of adherence to ART in India (8 studies; 1666 participants)^88^, found similar estimates for other low/middle income regions (77%; 95% CI: 68–85; *I* ^*2*^ **= **98.4% and 70%; 95% CI: 59–81, *I* ^2^ **= **96.3% respectively) than that found by our study. Pooled proportion of adherence has also been estimated by other researchers for North America (55%)^87^; Spain (55%)^89^; worldwide (62%)^90^; and for high‐risk subgroups living with HIV such as drug users (60%)^91^, pregnant women (73.5%)^92^; female sex workers (76%) 93, adolescents (62%) 20, prisoners (54.6%) 94 and different high‐risk populations living with HIV in China (77.61%) 95.

Our results show that, when assessing adherence, depending on the time‐frame for recall, different results might be achieved. Our results point to higher adherence (80%) in the shortest time frame and lower adherence (55%) in the longer time‐frame. These findings are consistent with the meta‐analysis of adherence among HIV‐positive drug users, conducted by Malta et al. (2010) 91, where the pooled estimate for the shortest period was higher (71%) than the pooled estimated for the intermediate period (54%). Moreover, and again, similarly to Malta et al. (2010) 91, the shortest time frame also yielded a less heterogeneous estimate of adherence. Taken together, these findings suggest that a shorter time frame might yield estimates that are less prone to recall bias and thus more accurate. Conversely, a longer time interval increases the chances of adherence issues, what may not be observed using a shorter time frame. That said, which adherence time frame best predicts virological failure is less known, with a few studies suggesting that the impact of the time frame might be minimal 21, 96.

There were no significant differences in the pooled adherence among different optimal adherence thresholds (<94%, 95%, 100%), which was similar to the findings of Ortego et al. (2011) 90. Moreover, in a recent meta‐analysis conducted by Bezabhe et al. (2016)^21^, there were no significant differences in the pooled odds ratios for virological failure among different optimal adherence thresholds (OR _≥98–100%_ **= **0.54, 95% CI: 0.29–1.00, *I* ^2^= 85%; OR _≥95%_ **= **0.34, 95% CI: 0.24–0.47, *I* ^2^ **= **92; OR _≥80–90%_ **= **0.34, 95% CI: 0.23–0.51, *I* ^2^= 0%) showing that irrespective of the cutoff point, optimal adherence to ART was associated with positive clinical outcomes.

As per Marmot, health inequalities are perhaps the most damning indictments of social and economic inequalities 97. Similar social gradients in health can be observed if we stratify the population by region, country or income. Social determinants of health can greatly affect adherence to ART. The proportion of people who reported optimal adherence to ART varied according to the country's income level, HDI and GNI per capita. We found that in studies from lower income countries the pooled proportion of adherence was higher than in studies from middle income countries. HDI and GNI per capita followed the same trend. Studies in countries with a lower HDI and GNI per capita had higher proportions of adherence than studies in countries with a higher HDI and GNI per capita. These findings are consistent with previous meta‐analysis: Uthman et al. (2014) 98 found that the proportion of PLHIV who achieved good adherence was significantly higher in lower‐income countries (86%) compared to higher‐income countries (67.5%; *p *<* *0.05). Although our findings suggest that less developed countries can achieve the same or higher level of adherence than more developed ones, huge differences between populations and availability of healthcare may have been included in each setting (selection bias). It is possible that in lower‐income countries participation in a research study provides better health care (e.g. ideal trial conditions and adherence counselling). In higher‐income countries, where people may have access to a better public service or can afford for a private service, other factors beyond poverty can affect ART adherence (e.g. depression and substance misuse). Bezabhe et al. (2016) 21 when exploring the impact of adherence on virological failure found that the pooled odds ratio for virological suppression among those with optimal adherence compared to suboptimal adherence for countries with low HDI (0.50; 95% CI: 0.35–0.72) was lower than for countries with very high‐HDI (0.23; 95% CI: 0.15–0.33). Suggesting that although adherence may be similar or higher, the expected effect of adherence on virologic suppression is lower in low/middle income countries. A possible explanation might be the lack of consistent virological monitoring 99 with patients adhering to non‐suppressive treatment.

Measures of adherence include individual self‐report, pharmacy records, pill counts, electronic measurement devices, therapeutic drug concentrations and clinical outcomes. The easiest and therefore most frequently used method is patient self‐report. Self‐report questions consider the number or frequency of doses missed/taken, or simply asks individuals to rate their adherence level, always considering a specific time period. Questionnaires frequently use a Likert‐type scale as the response format (often five‐point ordered response ranging from the most positive to the most negative response to a statement). Although individual self‐report can be inexpensive, easy to administer, and accurately identify medication‐taking behaviour 100, they may also overestimate adherence due to social desirability (i.e. respondents answer questions in a way that will be viewed favourably by clinicians) and recall biases 101. These biases may have impacted our results, because patient's self‐reported adherence was the most common method for assessing adherence in this meta‐analysis. Self‐report questionnaires, which have a reasonable predictive power, are useful for resource‐limited clinical settings. The ACTG Adherence Questionnaire was the most extensively used instrument. It is a 5‐item self‐report measure, but frequently, only the first item (four‐day recall of how many doses have been missed) is used in clinical setting. The CEAT‐VIH was the second most used self‐report instrument. The CEAT‐VIH is a short (20 items) multidimensional self‐report instrument to measure adherence, available in six languages: English, European Spanish and Latin American Spanish, European Portuguese, Brazilian Portuguese and Romanian. Although MEMS has been used as measure of choice to validate adherence measures such as patient's self‐report or pharmacy database (medication withdrawal or refill data), the cost of this device substantially impairs its widespread use. Clinical outcomes, is considered by some researchers as one of the best measures of a patient's adherence behaviour, but the use of clinical outcomes as a proxy of adherence can always be biased by the presence of any patient‐ or disease‐related factor. Each adherence measurement strategy has strengths and weaknesses. The best measurement strategy for clinical practice should take in consideration the setting, the population, and most importantly have acceptable reliability and validity.

We found that the assessment of the quality of the published studies was sometimes challenging. Many studies did not report relevant methodological details about the assessment of adherence, making it difficult to judge the strength of their findings. The absence of these data may have introduced a possible bias in the results (data retrieval bias). To promote improvement in the quality of measurement of medication adherence in research Williams et al. 102 have proposed a set of best practices for conducting adherence measurement. For studies using self‐report, for example, it is recommended the use of an instrument and method of administration that demonstrate both concurrent and predictive validity. When using a new instrument, it needs to be validated in a pilot test. When a scale is used to measure adherence, it needs to be culturally sensitive, worded clearly, and subjects need to know how to respond to the scaling response options with little difficulty. In addition to their recommendations, and to improve also the reporting of adherence measurement, we suggest that researchers clearly identify the instrument used, whether it was validated for use in the study population, when the assessment was carried out (date), the adherence recall time frame used, the adherence definition used (i.e. no. of pills taken/prescribed or instrument score), the optimal adherence cutoffs or thresholds adopted and ART used, which can greatly affect adherence to ART. Accurate assessment of adherence behaviour is essential for treatment planning while accurate reporting of adherence studies is essential for further advancement of the subject.

Identifying specific barriers for patients and implementing appropriate interventions to overcome them is extremely necessary to improve adherence. The social support was one of the factors associated with adherence in this systematic review and meta‐analysis. Social network and social influence, can provide a powerful approach for health behaviour change 103. Alcohol use was associated with nonadherence to ART in many studies. This association has been observed previously, where alcohol abuse can be higher in PLHIV than in the general population, and may lead to medical and psychiatric complications, poor adherence and poorer treatment outcomes 104. In addition, alcohol use is associated with intravenous drug use and risky sexual behaviour, major modes of HIV transmission 104. Unemployment was another barrier to nonadherence in LAC countries. This association exists globally, and was recently estimated with a pooled odds ratio of 1.27 (95% CI: 1.04–1.55) in a meta‐analysis carried out by Nachega et al. (2015) 105. Reporting of traditional barriers to ART such as toxicity and pill burden has reduced over time since current ART regimens are simpler and better tolerated. Consequently, the primary individual barriers to adherence have changed. In a recent meta‐analysis conducted by Shubber et al. (2016) 106, individual barriers most frequently reported by patients included forgetting, being away from home, and a change to daily routine. Depression, alcohol/substance misuse, stigma, feeling sick, health service‐related barriers (i.e. distance to clinic) and stock outs were less frequently reported. In this systematic review and meta‐analysis studies reported many traditional barriers, in particular those related with alcohol and substance misuse. The presence of so many traditional barriers may be indictments that regimens in use in LAC are not aligned with WHO, PAHO and other agencies efforts to optimize drug regimens. In 2012, 4% of the patients of the region, approximately 30,000 patients out of the 725,000 that receive ART, were being treated with obsolete or inappropriate ARVs 107. Also, in many countries the number of regimens in use still exceeds 15**–**20 108.

This study has important limitations worth noting. Meta‐analysis of observational studies, unlike randomized controlled trials, are prone to biases including confounding. The high risk of selection biases within included studies may be a function of their observational design. Moreover, we found high heterogeneity among the studies indicating that adherence varied significantly across studies, possibly due to different populations, different thresholds, different time frames and methods of measurement. Accordingly, random effects models were chosen as our analytical framework to better accommodate the heterogeneity since it assumes that each study was drawn from populations that differ from each other in ways that could impact the proportion of adherents. Heterogeneity was not entirely explained by subgroup analysis. A meta‐regression could help investigate the sources of the heterogeneity across studies by studying the relationship between study‐level characteristics and adherence to ART, but it was not performed in this study due to discrepancies associated with reporting of participants’ characteristics. For example, we were not able to evaluate a possible relationship between adherence and different regimens because this information was rarely available among the included studies. Another limitation is that most of the studies included in this meta‐analysis used a cross‐sectional design, making it difficult to determine causal relationships between level of adherence and other factors. Lastly, we did not include grey literature and therefore may have missed studies that were relevant to our research question during the literature search. However, the inclusion of grey literature may itself introduce biases if the studies found were not representative of all unpublished studies. Though, our results must be interpreted with caution, considering our assumptions and limitations, meta‐analyses are still the preferred methodology in providing a qualitative interpretation of the results 109.

## 5 Conclusions

In conclusion, our study suggests that PLHIV in LAC can achieve comparable adherence levels to other populations of developing regions though it may be below the sufficient levels required for successful long‐term viral load suppression. Monitoring ART adherence is critical to provide information about possible causes of virological failure in LAC, where viral load testing is often carried out less frequently than regional guidelines recommend 99. We encourage initiatives for improving ART adherence that consider the social determinants of health inside each context, involving community‐based organizations and social participation to address the huge socio‐economic disparities and the health inequities present across and within LAC countries.

## Competing interests

The authors declare that they have no competing interests.

## Authors' contributions

All authors contributed extensively to the work presented in this paper. JMC, TST, PML conceived the study. JMC, TST and PML created and performed the literature search strategy, JMC built the data extraction file, JMC and TST performed the data extraction and PML supervised the process. PML and LEC performed the statistical analyses, and all authors interpreted the data. JMC drafted the manuscript, and all the other authors contributed substantially to the writing and revising of the manuscript. All authors have read and approved the final version of the manuscript.

## Supporting information


**Additional file 1** File in format docx. PubMed search strategy for studies of adherence to antiretroviral therapy for HIV/AIDS in Latin America and the Caribbean.
