# Supplementary material for: Adherence to antiretroviral therapy for HIV/AIDS in Latin America and the Caribbean: Systematic review and meta‐analysis
Source: J Int AIDS Soc. 2018 Jan 22;21(1):e25066. doi: 10.1002/jia2.25066 (PMC5810329; doi:10.1002/jia2.25066)
Supplement: Supplementary file 1 — Additional file 1 File in format docx. PubMed search strategy for studies of adherence to antiretroviral therapy for HIV/AIDS in Latin America and the Caribbean. [file JIA2-21-e25066-s001.docx]

**Additional file 1**

**PubMed search strategy for studies of adherence to antiretroviral therapy for HIV/AIDS in Latin America and the Caribbean**

| 1 | Adherence[Title/Abstract] OR adherence [MeSH Terms] OR nonadherence[Title/Abstract] OR nonadherence[MeSH Terms] OR non-adherence[Title/Abstract] OR non-adherence[MeSH Terms] |
| --- | --- |
| 2 | HIV[Title/Abstract] OR HIV[MeSH Terms] OR AIDS[Title/Abstract] OR AIDS[MeSH Terms] OR PLHIV[Title/Abstract] OR PLHIV[MeSH Terms] |
| 3 | Antiretroviral[Title/Abstract] OR Antiretroviral[MeSH Terms] OR Antiretrovirals[Title/Abstract] OR Antiretrovirals[MeSH Terms] OR HAART[Title/Abstract] OR HAART[MeSH Terms] OR ART[Title/Abstract] OR ART[MeSH Terms] |
| 4 | "Latin America"[Title/Abstract] OR "Latin America"[MeSH Terms] OR Latinoamerica[Title/Abstract] OR Latinoamerica[MeSH Terms] OR Latin*[Title/Abstract] OR Latin*[MeSH Terms] OR "Central America"[Title/Abstract] OR "Central America"[MeSH Terms] OR Centroamerica[Title/Abstract] OR Centroamerica[MeSH Terms] OR "Meso america"[Title/Abstract] OR "Meso america"[MeSH Terms] OR "Middle America"[Title/Abstract] OR "Middle America"[MeSH Terms] OR mesoamerica[Title/Abstract] OR mesoamerica[MeSH Terms] OR "South America"[Title/Abstract] OR "South America"[MeSH Terms] OR Sudamerica[Title/Abstract] OR Sudamerica[MeSH Terms] OR "America del sur"[Title/Abstract] OR "America del sur"[MeSH Terms] OR Caribbean[Title/Abstract] OR Caribbean[MeSH Terms] OR Caribe[Title/Abstract] OR Caribe[MeSH Terms] OR Argentina[Title/Abstract] OR Argentina[MeSH Terms] OR Argentin*[Title/Abstract] OR Argentin*[MeSH Terms] OR Bolivia*[Title/Abstract] OR Bolivia*[MeSH Terms] OR Brazil*[Title/Abstract] OR Brazil*[MeSH Terms] OR Brasil*[Title/Abstract] OR Brasil*[MeSH Terms] OR Colombia*[Title/Abstract] OR Colombia*[MeSH Terms] OR Chile*[Title/Abstract] OR Chile*[MeSH Terms] OR Ecuador*[Title/Abstract] OR Ecuador*[MeSH Terms] OR Guiana[Title/Abstract] OR Guiana[MeSH Terms] OR Guyana[Title/Abstract] OR Guyana[MeSH Terms] OR Guayana[Title/Abstract] OR Guayana[MeSH Terms] OR Paraguay*[Title/Abstract] OR Paraguay*[MeSH Terms] OR Peru*[Title/Abstract] OR Peru*[MeSH Terms] OR Suriname[Title/Abstract] OR Suriname[MeSH Terms] OR Surinam*[Title/Abstract] OR Surinam*[MeSH Terms] OR Uruguay*[Title/Abstract] OR Uruguay*[MeSH Terms] OR Venezuela[Title/Abstract] OR Venezuela[MeSH Terms] OR Venez*[Title/Abstract] OR Venez*[MeSH Terms] OR Belize*[Title/Abstract] OR Belize*[MeSH Terms] OR "Costa Rica"[Title/Abstract] OR "Costa Rica"[MeSH Terms] OR "Costa Ric*"[Title/Abstract] OR "Costa Ric*"[MeSH Terms] OR "El Salvador"[Title/Abstract] OR "El Salvador"[MeSH Terms] OR Guatemala[Title/Abstract] OR Guatemala[MeSH Terms] OR Guatemal*[Title/Abstract] OR Guatemal*[MeSH Terms] OR Honduras[Title/Abstract] OR Honduras[MeSH Terms] OR Hondur*[Title/Abstract] OR Hondur*[MeSH Terms] OR Panama[Title/Abstract] OR Panama[MeSH Terms] OR Panam* [Title/Abstract] OR Panam*[MeSH Terms] OR Mexico[Title/Abstract] OR Mexico[MeSH Terms] OR Mexic*[Title/Abstract] OR Mexic*[MeSH Terms] OR Cuba*[Title/Abstract] OR Cuba*[MeSH Terms] OR "Dominican Republic"[Title/Abstract] OR "Dominican Republic"[MeSH Terms] OR "Republica Dominicana"[Title/Abstract] OR "Republica Dominicana"[MeSH Terms] OR Dominic*[Title/Abstract] OR Dominic*[MeSH Terms] OR Haiti*[Title/Abstract] OR Haiti*[MeSH Terms] OR Jamaica[Title/Abstract] OR Jamaica[MeSH Terms] OR Jamaic*[Title/Abstract] OR Jamaic*[MeSH Terms] |
| 5 | No. 1 and 2 and 3 and 4 |
